# A Covalent 3D CNT@rGO Nano‐Hybrid for High‐Efficiency Conductivity in Lithium‐Ion Batteries

**DOI:** 10.1002/advs.202504721

**Published:** 2026-01-30

**Authors:** Junwen Tang, Jingbo Pang, Jie Wang, Huiming Liang, Ao Du, Long Kuang, Xiaoming Cai, Ming Qin, Cuixia Yan, Wu Zhou, Jinming Cai

**Affiliations:** ^1^ Faculty of Materials Science and Engineering Kunming University of Science and Technology Kunming Yunnan P. R. China; ^2^ School of Physical Sciences University of Chinese Academy of Sciences Beijing P. R. China; ^3^ Guangdong Morion Nanotechnology Co., Ltd Guangdong P. R. China; ^4^ Faculty of Mechanical and Electrical Engineering Kunming University of Science and Technology Kunming Yunnan P. R. China; ^5^ Southwest United Graduate School Kunming P. R. China

**Keywords:** CNT@rGO, covalent connection, high yield, multiple conductive pathways

## Abstract

The limitations of conventional conductive agents in lithium‐ion batteries, such as carbon black and graphite flakes, have driven the search for high‐performance alternatives. Carbon nanotubes (CNTs) and graphene offer exceptional conductivity and lower dosage requirements, but face challenges related to high costs and complex fabrication processes. Herein, we develop a facile and cost‐effective one‐step chemical vapor deposition (CVD) strategy to achieve ultrahigh‐yield CNT growth (7692.31%) on reduced graphene oxide (rGO), constructing a covalently integrated 3D CNT@rGO composite. When deployed as a conductive agent in LiFePO_4_ cathodes, the 3D architecture establishes multidirectional conductive networks that facilitate unimpeded electron/ion transport during electrochemical reactions. This results in significantly enhanced rate capability across 1‐6 C rates and exceptional cycling stability with 96.32% capacity retention after 300 cycles at 1 C. The synergistic attributes—including multidimensional conduction pathways, minimal catalyst residue (0.52%), and homogeneous dispersion—collectively provide an efficient and economical solution for next‐generation battery technologies. This work paves the way for scalable battery technologies utilizing high‐performance carbon‐based conductive agents.

## Graphical abstract

A facile one‐step CVD strategy achieves high‐yield (7692.31%) growth of CNTs on rGO, forming a covalently bonded 3D CNT@rGO hybrid. This structure enables multidirectional conductive networks in LiFePO_4_ cathodes, delivering superior rate performance, high ICE (99.88%), and 96.32% capacity retention after 300 cycles at 1 C.

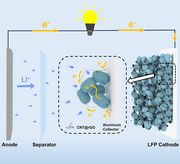

## 1 Introduction

The accelerated development of electric vehicles has led to heightened market expectations for lithium‐ion batteries, particularly with regard to safety, energy density, and rate performance [1, 2, 3]. LiFePO_4_ stands out among cathode materials owing to its excellent safety and cyclability, rendering it promising for electric vehicles. However, its low electronic conductivity (10^−^^9^–10^−^^1^ ^0^ S cm^−^^1^) and slow Li^+^ diffusion (∼10^−^^1^ ^4^ cm^2^ s^−^^1^) lead to pronounced polarization and capacity fading at high rates, limiting its use in high‐power applications [4, 5, 6, 7]. Conducting agents are indispensable components that enhance electrode conductivity and stabilize battery performance [8]. Among various conductive materials, single‐walled carbon nanotubes (SWCNTs) are regarded as optimal due to their excellent electrical conductivity and mechanical strength. However, their complex preparation process and elevated manufacturing cost hinder their extensive utilization [9].

In order to resolve the issues associated with SWCNTs, researchers have been exploring potential alternatives. Graphene and multi‐walled carbon nanotubes (MWCNTs) have been identified as promising candidates due to their lower preparation cost compared to SWCNTs and their excellent conductivity [10, 11, 12]. However, both materials exhibit challenges related to poor dispersion and a single dimension of electron transport. It is imperative to achieve uniform dispersion of the conductive agent to establish a robust conductive network for the electrode, as this is crucial for effective electron conduction and ensuring the overall performance of the electrode [13, 14]. To address this challenge, researchers have modified the material structure through in situ growth, doping, and compounding. Gupta et al. prepared multilayered PLA/Lithium Iron Phosphate/Carbon Nanotubes microarchitectural cathode materials using a 3D printing technique. The multilayered porous micro‐nanostructures significantly improved the ion and electron transport and exhibited remarkable charge/discharge characteristics with a capacity of 62 mAh g^−1^ at a 10 C rate [15]. In a separate study, Gao et al. synthesized lithium iron phosphate/carbon nanotube nanocomposites using a combination of in situ microwave plasma chemical vapor deposition and co‐precipitation. The open and highly conductive network established by the CNTs contributed to more efficient ion and electron conduction, with a discharge capacity of 126.1, 111.2, 99.5, and 71.3 mAh g^−1^ at 10 C, 20 C, 30 C, and 50 C rates, respectively, at high rates [16]. In the study by Chen et al., lithium iron phosphate was encapsulated with carbon nanotubes and amorphous carbon nanoshells by in situ autocatalysis. The initial capacity was recorded as 136.1 mAh g^−1^ at 1 C, and after 1000 cycles, the capacity was found to be 129.6 mAh g^−1^. This result indicates remarkable long cycling stability [17].

Existing studies have modified the active materials at the synthesis stage through in situ growth and other means, which can yield superior properties but are complex and costly, making them unsuitable for large‐scale use. In this study, a straightforward one‐step growth method is employed to achieve uniform dispersion of the catalyst copper nanoparticles, utilizing melamine as a monatomic dispersant. The growth of carbon nanotubes is subsequently initiated after loading on reduced graphene oxide, culminating in the production of CNT@rGO 3D nanoarchitecture materials. This method ensures a substantial yield of carbon nanotubes, amounting to 7652.31%, while maintaining a minimal catalyst residue of only 0.52%. The three‐dimensional nanoarchitecture of the material provides good electron transport properties in all three dimensions and facilitates uniform dispersion among the active materials, with an ICE of 99.88% at 0.1 C, and higher capacity retention and cycling stability than rGO, MWCNT, and rGO&MWCNT in 1‐6 C, with a capacity retention of 96.32% after 300 cycles at 1 C. When considered in conjunction with the material structure and the corresponding electrochemical performance tests, CNT@rGO, which was uniformly dispersed among LiFePO_4_ nanoparticles, played a beneficial bridging and structural stabilization role and constructed an effective conductive network.

## 2 Results and Discussion

### 2.1 Materials Preparation and Characterization

**FIGURE 1:**
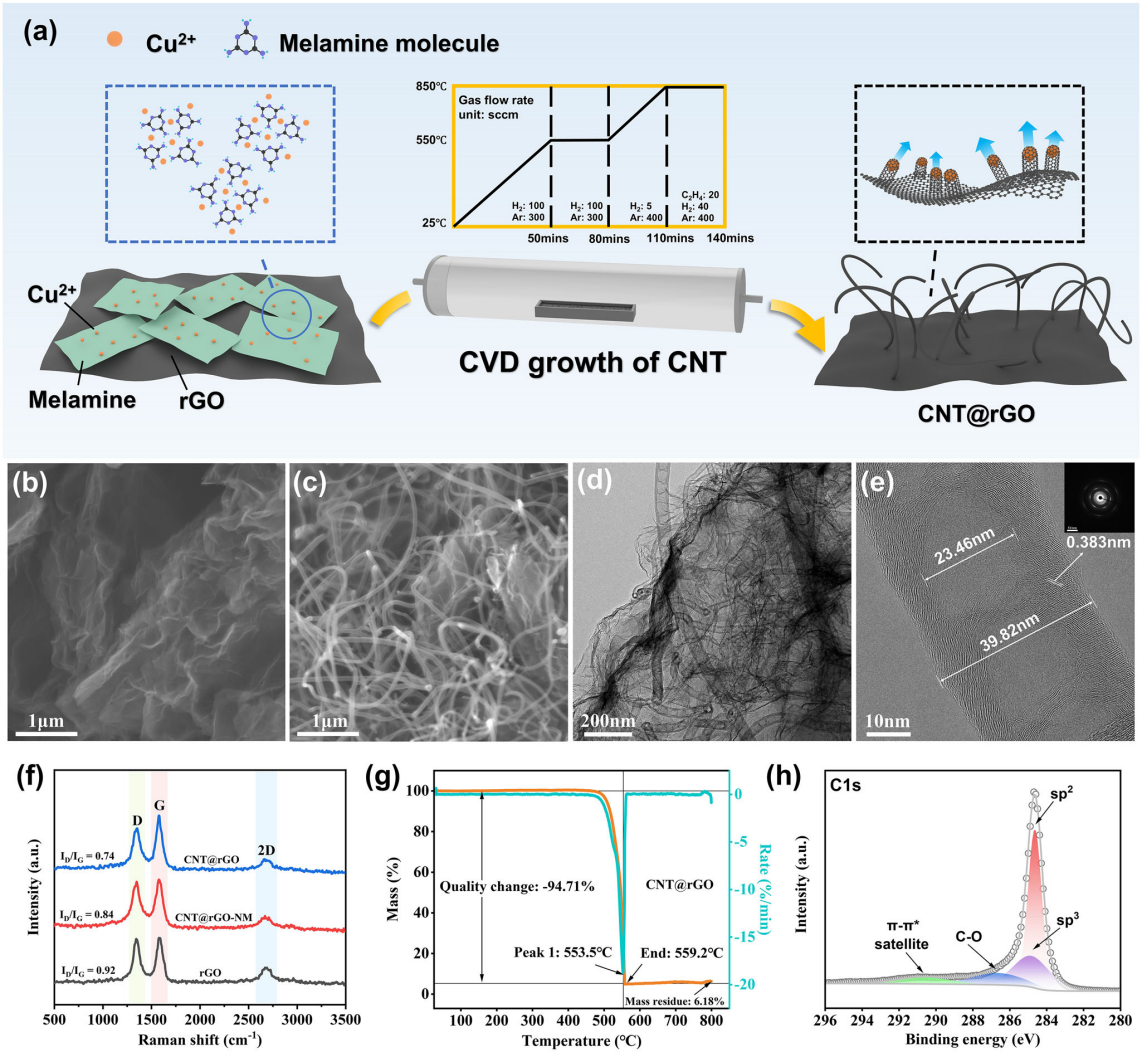
(a) Schematic of the preparation process of CNT@rGO. (b) SEM image of rGO. (c) SEM image of CNT@rGO. (d) HRTEM images of CNT@rGO. (e) HRTEM images of carbon nanotube morphology in CNT@rGO, insets are SAED images. (f) Raman spectra of rGO, CNT@rGO‐NM, and CNT@rGO. (g) TG and DTG curves of CNT@rGO. (h) XPS profiles of CNT@rGO C1s.

CNT@rGO was prepared using a simple two‐step method. In the first step, melamine was mixed with CuCl_2_‐2H_2_O in an ethanol solution and then dried. This process resulted in Cu being dispersed in the carbon‐nitrogen (CN) skeleton in the form of monatomic units, thereby constructing the supramolecular structure. In the second step, the mixture was then homogenously mixed with rGO powder and sent to a CVD tube furnace for a one‐step heat treatment. As the temperature increased, the melamine began to be thermally condensed, forming Cu‐g‐C_3_N_4_ with Cu atoms gradually. The formation of Cu‐C_3_N_4_ occurred as the temperature continued to rise. Concurrently, g‐C_3_N_4_ underwent gradual decomposition, resulting in the uniform dispersion of monatomic copper on the skeleton. This process subsequently led to the release and polymerization of copper in an orderly manner, culminating in the formation of homogeneous nanoparticles of a specific size. Following the introduction of C_2_H_4_ as a carbon source, the growth of carbon nanotubes was initiated, leading to the synthesis of nanomaterials with a 3D architecture of CNT@rGO upon cooling (Figure 1a).

Scanning electron microscope (SEM) was employed to obtain images of the original rGO and CNT@rGO samples; it reveals that carbon nanotubes exhibit dense and uniform growth on rGO (Figure 1b,c). Further magnification demonstrates the presence of a clear 3D structure (Figure S1a). To ascertain the effect of constructing a supramolecular structure on CNT growth, samples without melamine were labeled as CNT@rGO‐NM. Pristine rGO exhibited a folded morphology with lamellar stacking, and carbon nanotubes grew at some locations of the CNT@rGO‐NM, but in smaller amounts and with a larger difference in the morphology (Figure S2a,b). The superior morphology of CNT@rGO can be attributed to the atomic‐level dispersion of copper catalysts achieved by melamine. X‐ray diffraction (XRD) analysis of the mixture of melamine and CuCl_2_·2H_2_O (denoted as Melamine‐Cu) showed no characteristic peaks of copper when compared with the standard PDF card for copper (Figure S3a). In the Cu2p XPS spectrum, distinct satellite peaks of Cu(II) confirmed the presence of Cu^2^^+^ species (Figure S3b). In this system, copper ions from the metal salt act as hydrogen bond acceptors, while melamine serves as an organic ligand providing hydrogen bond donors. Through hydrogen bonding, a supramolecular compound is formed [18, 19]. The construction of this supramolecular architecture enables the ordered encapsulation and spatial isolation of copper ions by melamine molecules, resulting in molecular‐level dispersion and precise spatial confinement of copper. Concurrently, metal nanoparticles exhibit a significant size effect [20]. The effective dispersion of copper by melamine facilitates the subsequent formation of nano‐scale catalyst particles. This allows the carbon dissolution level of copper nanoparticles to reach a specific threshold at the carbon nanotube growth temperature, transitioning copper from a catalytically inert state to an active catalytic state [21]. Ultimately, carbon nanotube growth proceeds via a vapor–solid–solid (VSS) mechanism [22]. High‐resolution transmission electron microscopy (HRTEM) images demonstrate that the catalyst is located at the apex of the carbon nanotubes, with the base of the carbon tubes connected to the rGO via a junction (Figure S1b–d). The carbon nanotubes grown in CNT@rGO are of a dendritic nature, with a tube diameter distribution of 30–50 nm. The size of the agglomerates formed is determined by the size of the Cu nanoparticles after the pyrolysis of g‐ C_3_N_4_, and the dendritic morphology is due to the difference in the rate of diffusion of the carbon source on the surface of the catalyst and in the body of the large particles, which can be regarded as a series of seamlessly stacked cones. Furthermore, HRTEM observations revealed clear lattice stripes, arranged parallel to and at an angle to the growth axis, with uniform thickness on the outer wall of the tubes, and the thickness of the inner wall tapering from the bottom to the tip. In addition, selected electron diffraction spots indicate that the material possesses a well‐defined crystalline structure (Figure 1d,e).

In combination with the Raman spectra (Figure 1f), a more profound comprehension of the material's chemical composition and molecular structure is attained. The distinct D and G bands at 1350 and 1580 cm^−1^ are attributed to the disordered vibration of the structural defects and the in‐plane vibration of the sp^2^ carbon atoms, respectively. The pristine rGO I_D_/I_G_ ratio of 0.92 is attributable to defects present on the rGO lamellae, in addition to certain oxygen‐containing functional groups. The decrease in I_D_/I_G_ of both CNT@rGO‐NM and CNT@rGO with respect to the pristine rGO suggests that the growth of carbon nanotubes achieves a partial repair of the rGO defects. The lower degree of defects in CNT@rGO is attributed to the inhibition of amorphous carbon generation by NH_3_ produced by the thermal decomposition of melamine and the concomitant improvement in the morphology and structure of the carbon nanotubes. Thermogravimetric analysis (TG) was performed to gain a more accurate understanding of the intrinsic composition of the material. While the pristine rGO displays a single weight loss peak at 679.3°C, corresponding to the decomposition of sp^2^ C (Figure S4a), the CNT@rGO exhibits a single weight loss peak corresponding to the decomposition of the pure phase at 553°C. The residual mass of about 6% at the conclusion of the curve is attributed to the 3% intrinsic ash residue in the pristine rGO, respectively. 3% intrinsic ash residue and 3% oxidation products of the exposed copper catalyst (Figure 1g). CNT@rGO‐NM underwent two weight loss peaks corresponding to the decomposition of amorphous carbon and sp^2^ C at 563.1°C and 563.1°C, respectively (Figure S4b). The total X‐ray photoelectron spectroscopy (XPS) spectrum shows the atomic component occupancy of each element (Figure S5a). The high‐resolution XPS spectrum of C1s can be deconvoluted into four peaks at 284.6, 284.8, 286.6, and 290.68 eV, corresponding to the sp^2^, sp^3^, C─O, and π‐π* chemical states, respectively. The presence of the SP^3^ and C‐O peaks is attributed to defects in the material and to partial residues of oxygen‐containing functional groups (Figure S5b). Finally, the ICP test results indicated the presence of Cu in the CNT@rGO sample, with a content of 0.5217%, and the minimal catalyst content observed resulted in the elimination of a decontamination process, thereby preserving the integrity of the material structure.

### 2.2 Characterization and Calculations of Carbon Nanotube and rGO Connection Modes

**FIGURE 2:**
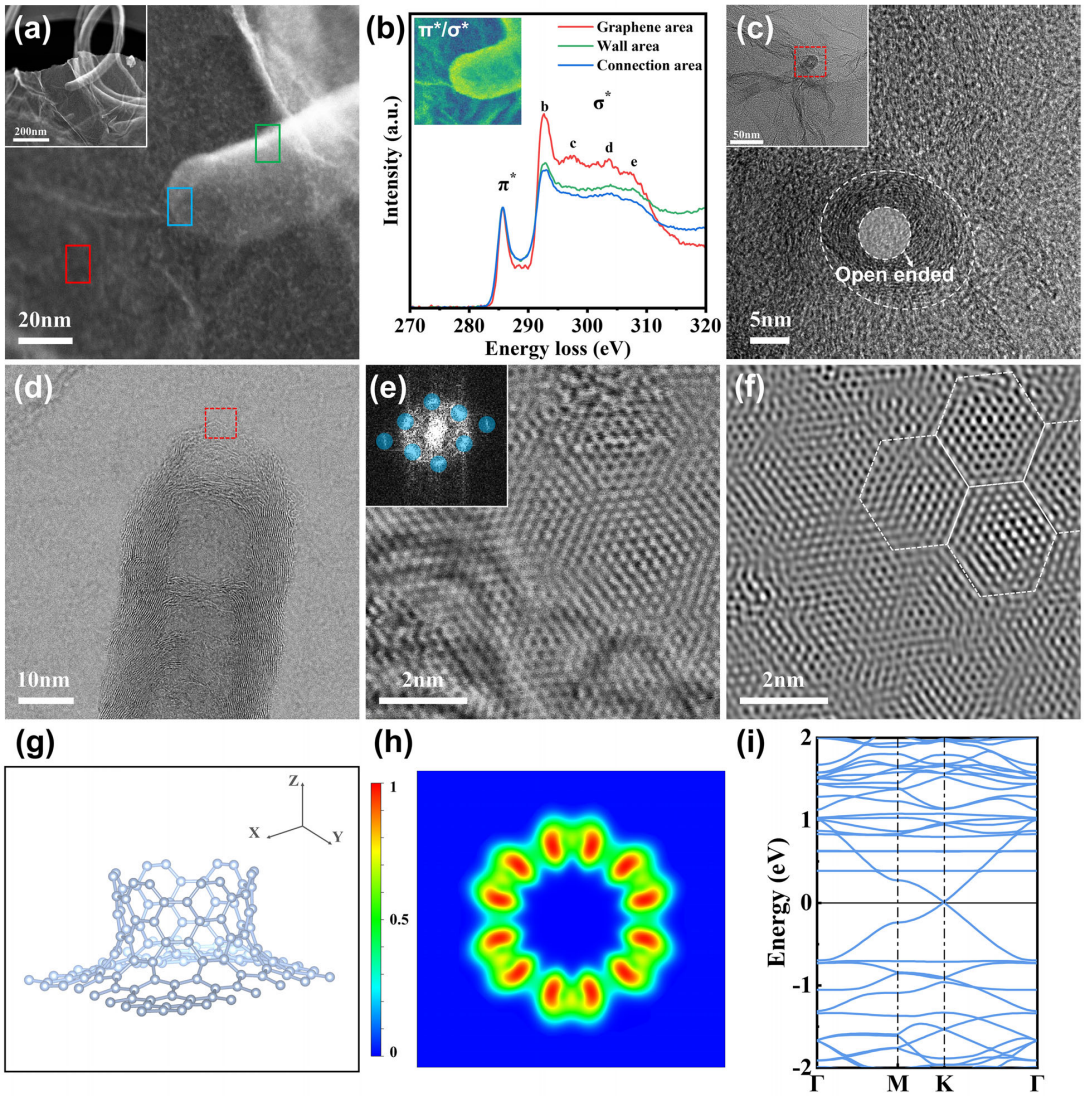
(a) SEI image of the CNT@rGO connection region. Rectangles with different colors represents different EELS acquisition regions. (b) EELS of CNT@rGO measured in different regions, inset is mapping of π*/σ* ratio intensity. (c) HRTEM image of the joint between CNT and rGO, inset is the HRTEM image of CNT on rGO slice. (d) BF STEM image of the connection area between CNT and rGO. (e) High resolution BF‐STEM image showing atom positions in connection area. Insert is the FFT of the image, and Blue circles show the positions of filters. Noise of the image can be excluded by doing inverse FFT of filter spots. (f) Inverse FFT image of (e). White dashed hexagons indicate the position of periodic moiré patterns, whose distribution is disrupted by the defects at the left bottom. (g) Modelling of CNT‐graphene connection. (h) The electron localization function in the XY plane (i) Energy band structure of the CNT‐graphene connection.

Scanning transmission electron microscopy (STEM) is employed to achieve a detailed characterization of the connection between CNT and rGO in CNT@rGO structures. The secondary electron image (SEI, Figure 2a) reveals a seamless connection between CNTs and rGO. Electron energy loss spectroscopy (EELS) analysis further demonstrates distinct atomic bonding characteristics in the rGO, connection, and wall regions (Figure 2b). Specifically, rGO exhibits a higher σ*/π* ratio compared to CNT, as the σ bonds in rGO are more aligned with the direction of momentum transfer, which is perpendicular to the beam incidence direction [23, 24, 25]. Within the CNT structure, the connection region shows a lower σ*/π* ratio than the wall region, attributed to its orientation being closer to perpendicular to the momentum transfer direction, thereby reducing the excitation of in‐plane σ‐bonding electrons. The π*/σ* intensity mapping corroborates this observation, showing a consistently lower σ*/π* ratio across the interface region. An open‐ended structural feature, indicative of covalent bonding between CNTs and rGO, is observed when the electron beam aligns with the CNT position (Figure 2c). High‐resolution bright‐field (BF) STEM images provide direct atomic‐scale insights into the interface region (Figure 2d,e). Noise‐filtered images reveal a certain degree of interfacial defects within the interface, as evidenced by the disruption of the periodic moiré pattern (Figure 2f). Further analysis shows that the defects are 7‐membered rings. (Figure S6a–d). This is consistent with the theoretical modeling, which shows the presence of 7‐membered ring defects at the connection region (Figure 2g).

**FIGURE 3:**
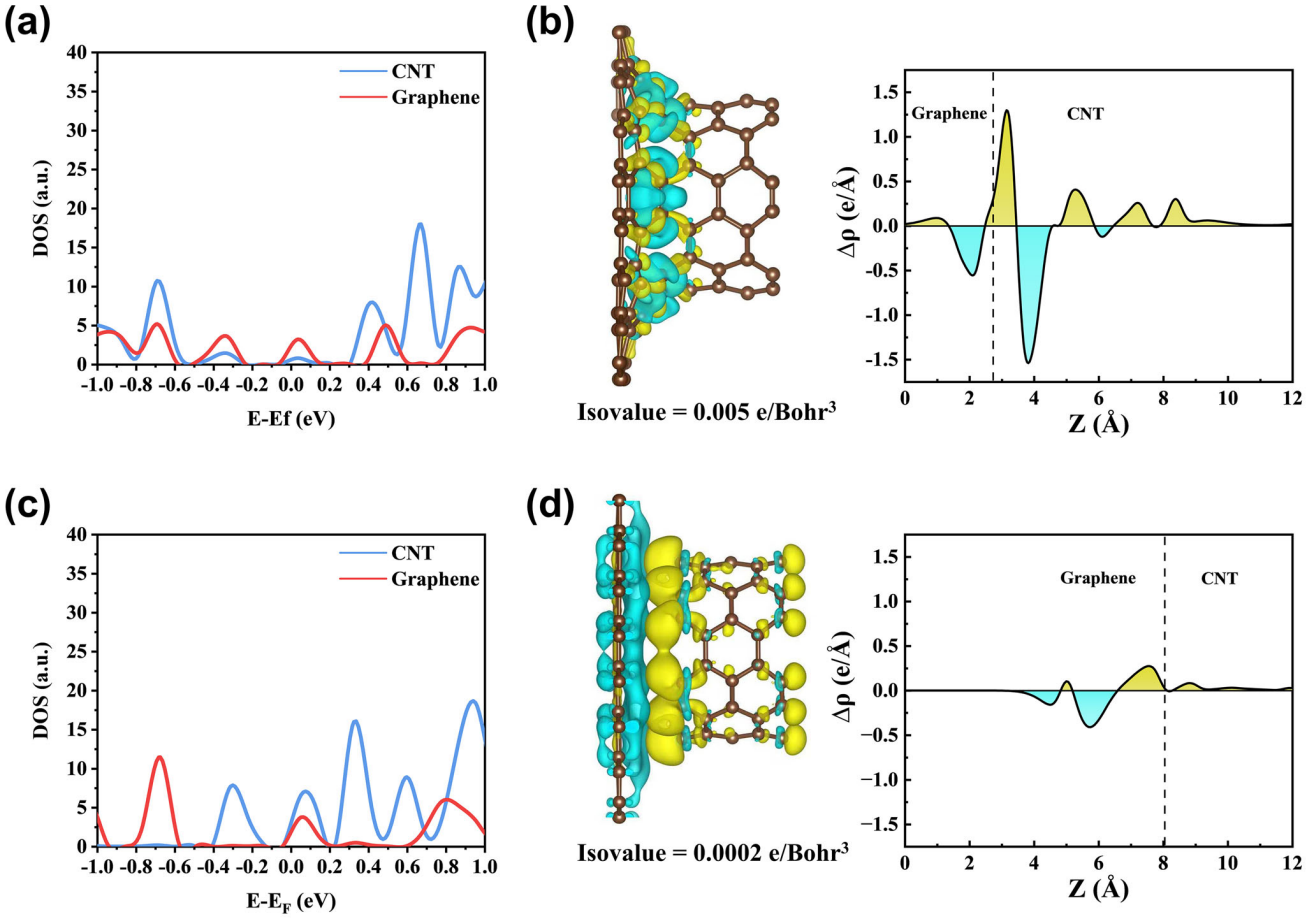
(a) PDOS of covalently bonded carbon nanotube‐graphene heterostructure; (b) PDOS of van der Waals‐coupled carbon nanotube‐graphene heterostructure; (c) Differential charge density map of carbon nanotube–graphene covalent connection; (d) Differential charge density map of carbon nanotube–graphene van der Waals connection.

To investigate the potential advantages of covalent bonding between carbon nanotubes and graphene, we compared this configuration with a van der Waals‐coupled CNT/graphene system. The electronic transport properties of both systems were evaluated through band structure and charge density difference analyses. Band structure calculations reveal the presence of Dirac cones in both covalently bonded and van der Waals‐coupled systems. In the covalent system (Figure 2h,i), the Dirac cone arises from covalent interactions at the interface, as evidenced by overlapping peaks in the projected density of states (PDOS) near the interface (Figure 3a) [26]. These interactions promote the formation of new hybrid electronic states with continuous band dispersion. In contrast, no overlapping peaks are observed in the PDOS near the interface of the van der Waals heterostructure (Figure 3c), indicating that the Dirac cone arises primarily from band alignment rather than band hybridization (Figure S7). By performing linear fitting of the bands near the Dirac point and using the equation $V_{f}=\frac{1}{\hbar }\frac{\partial E}{\partial k}$, we calculated Fermi velocities of 3.22 × 10^5^ m/s along the x‐direction (K_x_) and 3.38 × 10^5^ m/s along the y‐direction (K_y_) for the covalent system. For the van der Waals system, the corresponding values have been determined to be 2.52 × 10^5^ m/s (K_x_) and 2.41 × 10^5^ m/s (K_y_). In the systems studied, the higher Fermi velocities indicate intrinsically lower interfacial resistance according to the format: [27, 28]

$$R_{\mathrm{sharvin}}=\frac{h}{2e^{2}}\frac{4\pi }{{\left(k_{F}a\right)}^{2}}\alpha \frac{1}{{v_{F}}^{2}}$$

in which *R*_sharvin_ and *v*_F_ denote the contact resistance and Fermi velocity, respectively.

Charge density difference analysis (yellow: accumulation; blue: depletion) further corroborates these results. Planar‐averaged charge density difference analysis revealed a positive Δρ (Δρ > 0) at the interface of the covalently bonded structure (Figure 3b), indicating significant electron cloud overlap and sharing due to covalent bond formation [29]. which facilitates efficient pathways for charge transport. This strong electronic coupling effectively maintains high carrier mobility [30], consistent with the calculated Fermi velocities. In contrast, the van der Waals structure exhibited Δρ values approaching zero at the interface (Figure 3d), suggesting negligible electron cloud overlap. This weak electronic interaction cannot support efficient transport channels, leading to increased effective carrier mass and reduced Fermi velocity. Consequently, charge transport must rely on quantum tunneling across a physical barrier, ultimately resulting in higher contact resistance.

The seamless covalent bonding between carbon nanotubes and graphene not only preserves the intrinsic chemical properties and local sp^2^ hybridization structure of both components but also significantly enhances the electrochemical performance of the composite system. Compared to physically contacted van der Waals interfaces, this configuration exhibits superior electron conduction capability. Its characteristic semi‐metallic nature provides a high density of mobile electronic states near the Fermi level [31, 32], predicting the exceptional electron transport properties of CNT@rGO.

Equally important is the 3D nanostructure presented by CNT@rGO, which markedly optimizes ion transport within the electrode. The covalently bonded carbon nanotubes not only effectively inhibit the restacking of graphene layers but also construct a highly open hierarchical pore system, forming a “3D continuous transport network” conducive to rapid ion diffusion [33]. This structure greatly enhances the power density and active material utilization of the electrode. Furthermore, this mechanically robust 3D conductive network effectively accommodates the volume changes of active materials during electrochemical cycling, thereby endowing the electrode with excellent cycling stability and high‐rate capability. Through the synergistic coupling of graphene and carbon nanotubes, CNT@rGO achieves an optimized balance among electron conduction, ion transport, and structural integrity, demonstrating significant potential as a high‐performance conductive agent.

### 2.3 Electrochemical Performance of CNT@rGO as a Conductive Agent

In order to evaluate the electrochemical performance of CNT@rGO as a conductive agent for the LiFePO_4_ positive electrode, a CR2032 button half‐cell was assembled with lithium foil as the counter electrode for performance testing. To represent the profile, the name of the conductive agent was used to correspond with the given electrode. Prior to the assembly of the battery, the pole piece resistivity of the different electrodes was first obtained using the four‐probe method. The results obtained are as follows: CNT@rGO has the lowest resistivity of all the other electrodes, with a value of 3.76 Ω‐cm (Figure S8).

**FIGURE 4:**
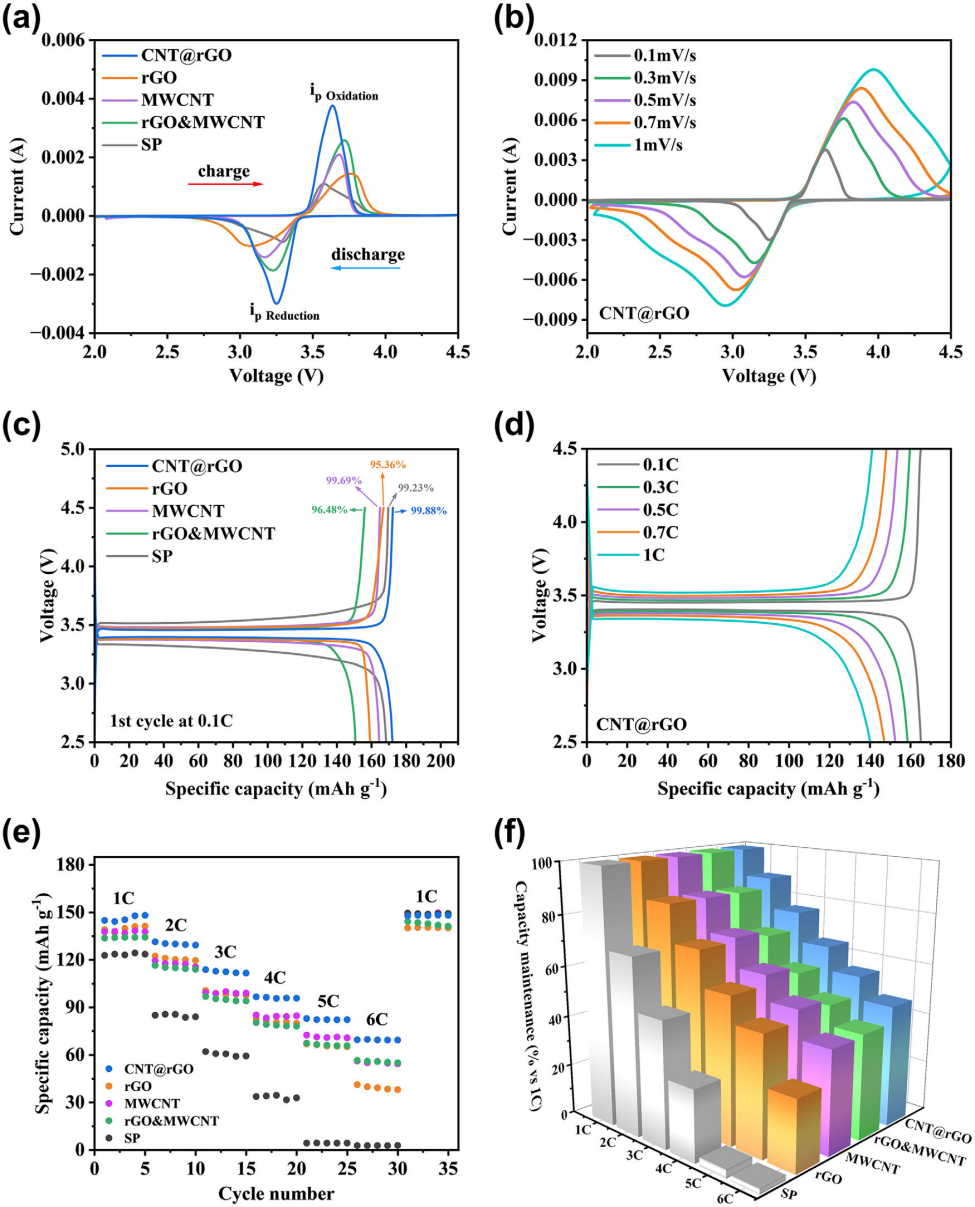
(a) CV curves of different electrodes at 0.1 mV/s sweep rate; (b) CV curves of CNT@rGO at different sweep rates; (c) Charge/discharge curves of different electrodes at 0.1 C for the first cycle; (d) Charge/discharge curves of CNT@rGO at different multiplicities; (e) Multiplicity test of different electrodes at 1‐6 C; (f) Different electrodes at 1‐6 C multiplicities 3D histogram of capacity retention at relative 1 C.

After cell assembly, cyclic voltammetry (CV) was performed to characterize the electrode reaction during charge/discharge processes. Additionally, the reversibility of the electrode reaction was determined. The peaks that appeared around 3.64 V during charging were attributed to the oxidation reaction of the LiFePO_4_ electrode, with larger peaks corresponding to a longer charging plateau. During discharging, the peaks around 3.25 V are identified as corresponding to the reduction reaction of the electrode (Figure 4a). The |i_po_/i_pr_| ratio reflects electrode reversibility, with values nearer to 1 indicating higher reversibility. In addition, the peak spacing has been demonstrated to reflect the degree of polarization of the electrode. The close proximity of |i_po_/i_pr_| of CNT@rGO to 1, in conjunction with its minimal peak spacing, signifies that the electrode is less polarized and possesses a higher degree of reversibility [34]. Furthermore, the CV tests at different sweep speeds demonstrated that the peak areas of the oxidation and reduction peaks varied to a similar extent with the increase in sweep speed (Figure 4b) [35]. This finding indicates that the CNT@rGO electrode exhibited excellent reversibility performance at varying multiplicities and possessed the lowest irreversible capacity in comparison to the other electrodes within the other groups (Figure S9a–d). Subsequently, the ICE of diverse electrodes at 0.1 C charging and discharging multiplicity was examined. The ICE of CNT@rGO, rGO, MWCNT, rGO&MWCNT, and SP was determined to be 99.88%, 95.36%, 99.69%, 96.48%, and 99.23%, respectively (Figure 4c). In the 0.1 C to 1 C multiplicity stage elevation tests, CNT@rGO exhibited a consistent and stable charging and discharging plateau, indicative of the stability and reversibility of the electrodes (Figure 4d).

Concurrently, the rate performance of the battery was evaluated. The 1‐6 C multiplicity test demonstrated that CNT@rGO exhibited the most stable cycling characteristics and the highest specific capacity at varying multiplicities (Figure 4e). The 3D histogram of capacity retention rate visually manifested the performance discrepancy among different conductive agents (Figure 4f). In order to analyze the reasons for the difference in electrode performance, we took cross‐sectional images of the pole pieces using SEM (Figure S10a–d); it is evident that CNT@rGO plays a pivotal role in encapsulation and bridging between LiFePO_4_ particles, exhibiting reduced agglomeration and stacking. Conversely, agglomeration of carbon nanotubes is prevalent in MWCNT and rGO&MWCNT, resulting in an increased number of tube contact points. This can impede current transmission, thereby reducing conductivity and adversely affecting the electrodes [36, 37]. Concurrently, a rapid capacity decay of rGO was observed at the 6 C rate, which is attributed to mechanical stress induced by repeated lithium‐ion insertion and extraction during high‐rate cycling. This stress leads to structural degradation of rGO, resulting in insufficient interlayer connectivity that causes electrode cracking. As a consequence, some active material particles lose electrical contact with the conductive network, become electrochemically isolated, increase the internal resistance, and contribute to irreversible capacity loss [38, 39]. In contrast, the covalently bonded 3D architecture of CNT@rGO provides enhanced mechanical stability during high‐rate charge and discharge processes. The well‐maintained electrode structure contributes to its superior rate capability (Figure S11).

**FIGURE 5:**
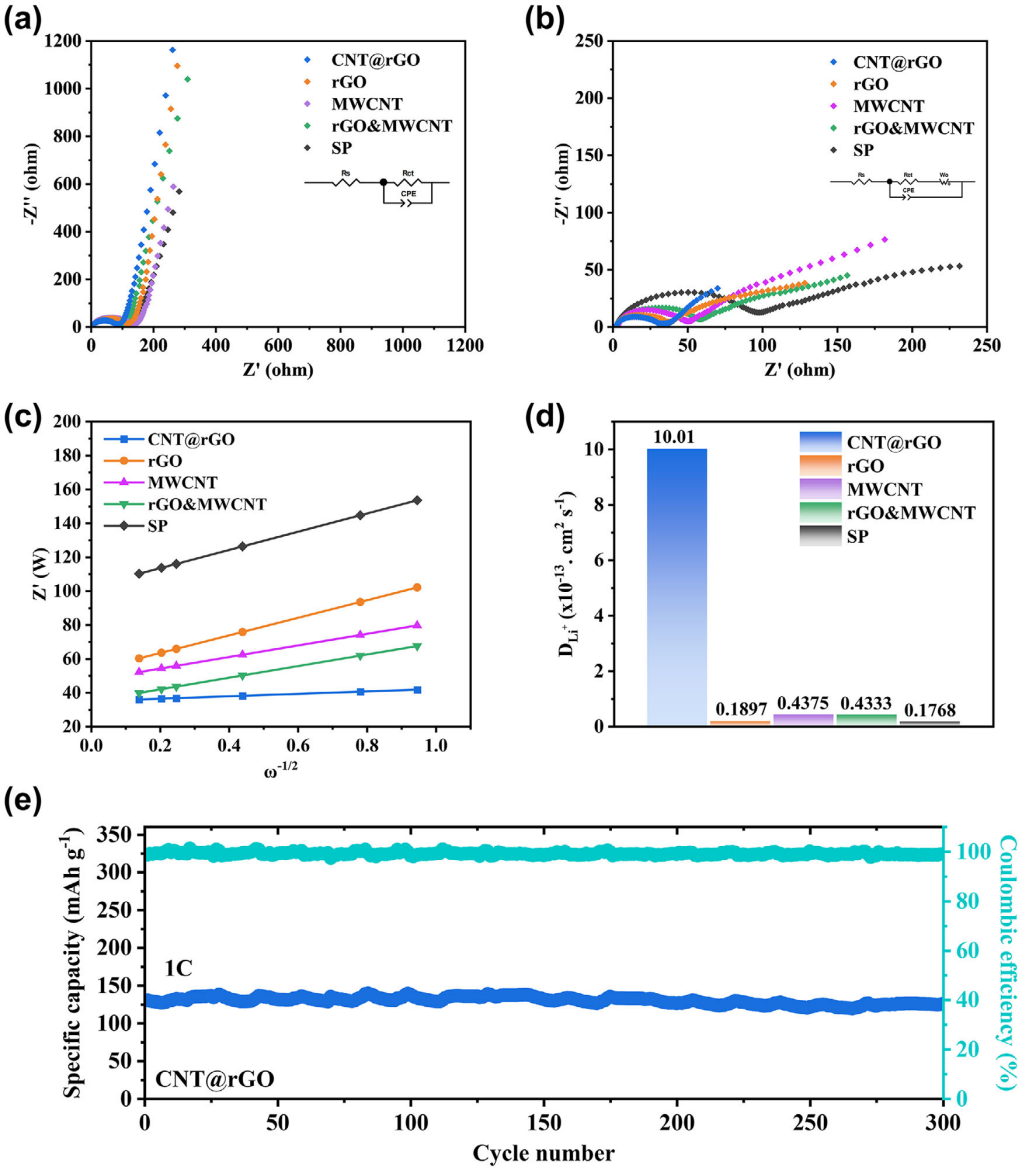
(a, b) Nyquist plots of different electrodes before and after cycling, and the inset is the fitted equivalent circuit diagram. (c) Z' versus ω‐1/2; (d) Li‐ion diffusion coefficients of different electrodes; (e) CNT@rGO tested at 1 C for 300 cycles.

In order to conduct a more in‐depth investigation into the effects of different conductive agents on the electrode electrochemical reactions, EIS tests were performed before and after the battery cycling (Figure 5a,b). In the high‐frequency region, the resistance of the CNT@rGO electrode for electron transfer, R_ct_ was measured to be 79.71 Ω before cycling and 29.78 Ω after cycling (Figure S12a,b). The straight line in the low‐frequency region is controlled by the diffusion of Li^+^. In order to verify the effect of different conductive agents on the diffusion of lithium ions, we fitted and merged the impedance data to plot Z' versus the angular frequency of the low‐frequency region between the inverse square root ω^−1/2^ (Figure 5c), where the slope of the straight line indicates the Warburg coefficient factor σ. The following equation is employed: [40]

$$D_{Li^{+}}=\frac{R^{2}T^{2}}{2A^{2}n^{4}F^{4}C^{2}\sigma^{2}}$$

The diffusion coefficient D_Li+_ is inversely related to the Warburg coefficient factor σ, with smaller slopes corresponding to larger Li‐ion diffusion coefficients. The calculation results demonstrate that CNT@rGO has the highest D_Li+_, which is consistent with the performance test (Figure 5d). Finally, 300 long cycle tests were performed at a 1 C charge/discharge rate, and it was found that CNT@rGO exhibited optimal cycling stability, with a residual specific capacity of 125.5 mAh g^−1^ at the end of the cycle, and a capacity retention of 96.32% (Figure 5e), compared to the other electrodes (Figures S13a,b and S14a,b). CNT@rGO plays a better role in the LiFePO_4_ particles between the bridging and structural stabilization, maintaining excellent electron and ion transport properties under long cycling. When combined with the results of material characterization and electrochemical analysis, the 3D conductive network architecture formed by CNT@rGO between the LiFePO_4_ active materials significantly improves the lower electronic and ionic conductivities, enhances the structural stability of the electrodes, and significantly improves the rate performance and cycling stability of the battery.

Finally, a comparative analysis is conducted on the cost and performance of CNT@rGO conductive agent material in relation to other prevalent conductive agent materials currently available on the market (Figure S15). Despite exhibiting slightly diminished performance in comparison to graphene and SWCNT, CNT@rGO demonstrates notable cost efficiency, indicating its considerable potential for practical applications in the future.

## 3 Conclusion

In summary, a melamine‐assisted monatomic dispersion of Cu nanoparticles on rGO enabled CVD growth of a 3D CNT@rGO nano‐architecture. The carbon nanotube yield was exceptionally high at 7652.31%, with minimal catalyst residue of only 0.52%. When applied to LiFePO_4_ cathode materials, the rate performance of batteries was superior to that of conventional conductive agents, with a capacity retention of 96.32% after 300 cycles at 1 C. The results indicate that CNT@rGO, as a carbon‐based conductive agent with a simple preparation process, low cost, high yield, and low catalyst residue, has high potential for application in LiFePO_4_ cathodes for lithium‐ion batteries, and it is expected to be prepared and applied on a large scale in the future.

## Funding

This work received financial support from the National Key R&D Program of China (No. 2024YFA1207800), the National Natural Science Foundation of China (No. 22372074), the CAS Project for Young Scientists in Basic Research(YSBR‐003), the Yunnan Fundamental Research Projects (202401AU070147, 202301BE070001‐026), the Major Basic Research Project of Science and Technology of Yunnan (202302AG050007), Yunnan Innovation Team of Graphene Mechanism Research and Application Industrialization (202305AS350017), and Graphene Application and Engineering Research Center of Education Department of Yunnan Providence (KKPP202351001).

## Conflicts of Interest

The authors declare no conflicts of interest.

## Supporting information




**Supporting File**: advs71974‐sup‐0001‐SuppMat.docx.

## Data Availability

The data that support the findings of this study are available from the corresponding author upon reasonable request.
