# Supplementary material for: A Covalent 3D CNT@rGO Nano‐Hybrid for High‐Efficiency Conductivity in Lithium‐Ion Batteries
Source: Adv Sci (Weinh). 2026 Jan 30;13(12):e04721. doi: 10.1002/advs.202504721 (PMC12948244; doi:10.1002/advs.202504721)
Supplement: Supplementary file 1 — Supporting File: advs71974‐sup‐0001‐SuppMat.docx. [file ADVS-13-e04721-s001.docx]

Supporting Information

**Covalently Integrated CNT@rGO for Superior Conductivity and Cycling Stability in Lithium-Ion Batterie**

*Junwen Tang^a#^, Jingbo Pang^b#^, Jie Wang^c#^, Huiming Liang^c^, Ao Du^a^, Long Kuang^a^, Xiaoming Cai^d^, Ming Qin^a^, Cuixia Yan^a^, Wu Zhou^b^, Jinming Cai^ace^**

*a Faculty of Materials Science and Engineering, Kunming University of Science and Technology, Kunming, Yunnan 650093, PR China*

*b School of Physical Sciences, University of Chinese Academy of Sciences, Beijing 100049, China*

*c Guangdong Morion Nanotechnology Co., Ltd, Guangdong 523815, PR China*

*d Faculty of Mechanical and Electrical Engineering, Kunming University of Science and Technology, Kunming, Yunnan 650500, PR China*

*e Southwest United Graduate School, Kunming, 650000, PR China*

**Corresponding author. Email: j.cai@kust.edu.cn*

*^#^* These authors contributed equally to this work.

Keywords: CNT@rGO, high yield, covalent connection, multiple conductive pathways

Experimental Methods

***Commercial Materials***

LiFePO₄ powder was purchased from Shenzhen KejingZhida Technology Co. Carbon black (Super P) and polyvinylidene fluoride (PVDF) were obtained from Dongguan Keruder Innovative Technology Co. Multi-walled carbon nanotubes (MWCNTs) were acquired from Shandong Tianyu Nano Technology Co. Graphene oxide (GO) was synthesized from natural graphite (500 mesh, Qingdao Tengshengda Carbon Machinery Co., Ltd.) using the modified Hummers method. Reduced graphene oxide (rGO) was prepared by heat-treating graphene oxide. Melamine and copper chloride dihydrate were purchased from Shanghai Aladdin Reagent Co. All materials were used without further purification.

***Preparation of CNT@rGO***

The specific synthesis of CNT@rGO materials can be described as follows: Melamine (0.3 g) and CuCl₂·2H₂O (0.0027 g) were dispersed in anhydrous ethanol at 80°C for mixing and stirring. After the ethanol had evaporated, the obtained powder was homogenously mixed with reduced graphene oxide powder (0.1 g). Subsequently, the uniformly mixed powder was loaded into a crucible and placed in a CVD tube furnace for the growth stage of carbon nanotubes. Initially, a gas mixture of 100 sccm hydrogen and 300 sccm argon was introduced to raise the temperature to 550°C, which was maintained for 30 minutes under the same gas flow rate. Then, the hydrogen flow rate was adjusted to 5 sccm, and the argon flow rate was increased to 400 sccm, while the temperature was further raised to 850°C. Upon reaching 850°C, the temperature was held for an additional 30 minutes, during which 20 sccm ethylene, 40 sccm hydrogen, and 400 sccm argon were introduced. After natural cooling to room temperature, the final product was obtained as a black, fluffy powder.

***Calculation of yield***

$$Y=\frac{m_{mix}-m_{catalyst}X}{m_{catalyst}X}\times100\%$$

The symbol 'Y' represents the yield. The term m_mix_ denotes the total mass of carbon nanotubes and residual catalyst in the product, while m_catalyst_ indicates the mass of catalyst prior to the reaction. The symbol 'X' is used to represent the relative percentage of the catalyst mass remaining after the reaction. This relative percentage is determined by analyzing the sample using Inductively Coupled Plasma Optical Emission Spectrometry (ICP-OES) after the reaction.

***Preparation of rGO&MWCNT***

The MWCNTs were compounded with rGO at a ratio of 4:6, with the resulting material designated CNT@rGO.

***Preparation of electrodes and cells***

The synthesized CNT@rGO material was used as a conductive agent for the LiFePO₄ positive electrode material. The powder was dissolved in N-Methyl-2-pyrrolidone (NMP) solvent and subjected to a high-pressure homogenization process for 10 minutes to obtain a CNT@rGO conductive agent slurry. Subsequently, LiFePO₄ (active material), polyvinylidene fluoride (PVDF), Super P, and the CNT@rGO conductive agent slurry were mixed at a weight ratio of 92:2:5:1 to prepare the electrode. The slurry was uniformly coated onto aluminum foil and dried in an air blast oven at 80°C for 1 hour, followed by vacuum drying for 12 hours. The dried electrodes were cut into discs with a diameter of 12 mm.

The assembly of the CR2032 coin cell was performed in an argon-filled glove box with H₂O and O₂ levels maintained below 0.01 ppm. The LiFePO₄ electrode containing the CNT@rGO conductive agent served as the working electrode, while lithium foil was used as the counter electrode. The electrolyte consisted of 1.0 M LiPF_6_ in a mixture of ethylene carbonate (EC) and diethyl carbonate (DEC) (v/v = 1:1), and a polypropylene (PP) membrane was used as the separator.

***Instrumental characterization***

X-ray diffraction (XRD) analysis was performed using a Rigaku Ultima IV diffractometer. Raman spectroscopy was conducted using a WITec alpha300R spectrometer, while thermogravimetric analysis (TGA) was carried out using a Netzsch TG 209 F3 thermogravimetric analyzer. X-ray photoelectron spectroscopy (XPS) measurements were obtained using a Thermo Scientific K-Alpha spectrometer. Field emission scanning electron microscopy (SEM) images were acquired using a JSM-7610F microscope at an accelerating voltage of 10 kV. Transmission electron microscopy (TEM) images were captured using a JEOL JEM-F200 microscope. Inductively coupled plasma (ICP) tests were performed using an Agilent ICP-OES 5800 spectrometer. Scanning transmission electron microscopy (STEM) images, secondary electron images (SEI), and electron energy loss spectra (EELS) were taken on an aberration-corrected JEOL GRANDARM2 scanning transmission electron microscope at 80 kV.

***Electrochemical and cell measurements***

The resistivity of the electrode was measured using a four-probe tester (Jinko Solar ST2263). Constant-current charge/discharge, rate capability, and long-cycle performance tests were conducted using a multi-channel battery tester (LAND CT2001A). Electrochemical impedance spectroscopy (EIS) measurements were carried out using an electrochemical workstation (Shanghai Chenhua CHI660E) in the frequency range of 0.1 Hz to 100 kHz.

*Calculation method*

The first-principles calculations, based on density functional theory (DFT), were carried out using the projector augmented wave (PAW) method in the VASP software package. The electron exchange-correlation potential was approximated using the generalized gradient approximation (GGA) with the Perdew–Burke–Ernzerhof (PBE) functional. The kinetic energy cutoff was set to 550 eV to enhance the plane wave basis set. The system optimization was considered complete when the total energy difference between two consecutive iterations was less than 0.001 eV, with the electronic convergence criterion set to 10^−8^ eV. The planar average differential charge density is calculated as follows: ∆ρ(z) = ∫ρ_nanotube-graphene_(x,y,z)dxdy − ∫ρ_nanotube_(x,y,z)dxdy − ∫ρ_graphene_(x,y,z)dxdy, where ρ_nanotube-graphene_(x,y,z), ρ_graphene_(x,y,z), and ρ_nanotube_(x,y,z) represent the charge densities at point (x,y,z) for the nanotube-graphene system, graphene, and nanotube.

**Table S1. Based on the nomenclature of different conductive agent electrodes and their corresponding percentage of each component**

|  | | LFP  (%) | | SP  (%) | | rGO  (%) | | MWCNT  (%) | | CNT@rGO  (%) | | PVDF  (%) | |
| --- | --- | --- | --- | --- | --- | --- | --- | --- | --- | --- | --- | --- | --- |
| SP | 92 | | 6 | | 0 | | 0 | | 0 | | 2 | |  |
| MWCNT | 92 | | 5 | | 0 | | 1 | | 0 | | 2 | |  |
| rGO&MWCNT | 92 | | 5 | | 0.6 | | 0.4 | | 0 | | 2 | |  |
| rGO | 92 | | 5 | | 1 | | 0 | | 0 | | 2 | |  |
| CNT@rGO | 92 | | 5 | | 0 | | 0 | | 1 | | 2 | |  |
|  | |  | |  | |  | |  | |  | |  | |


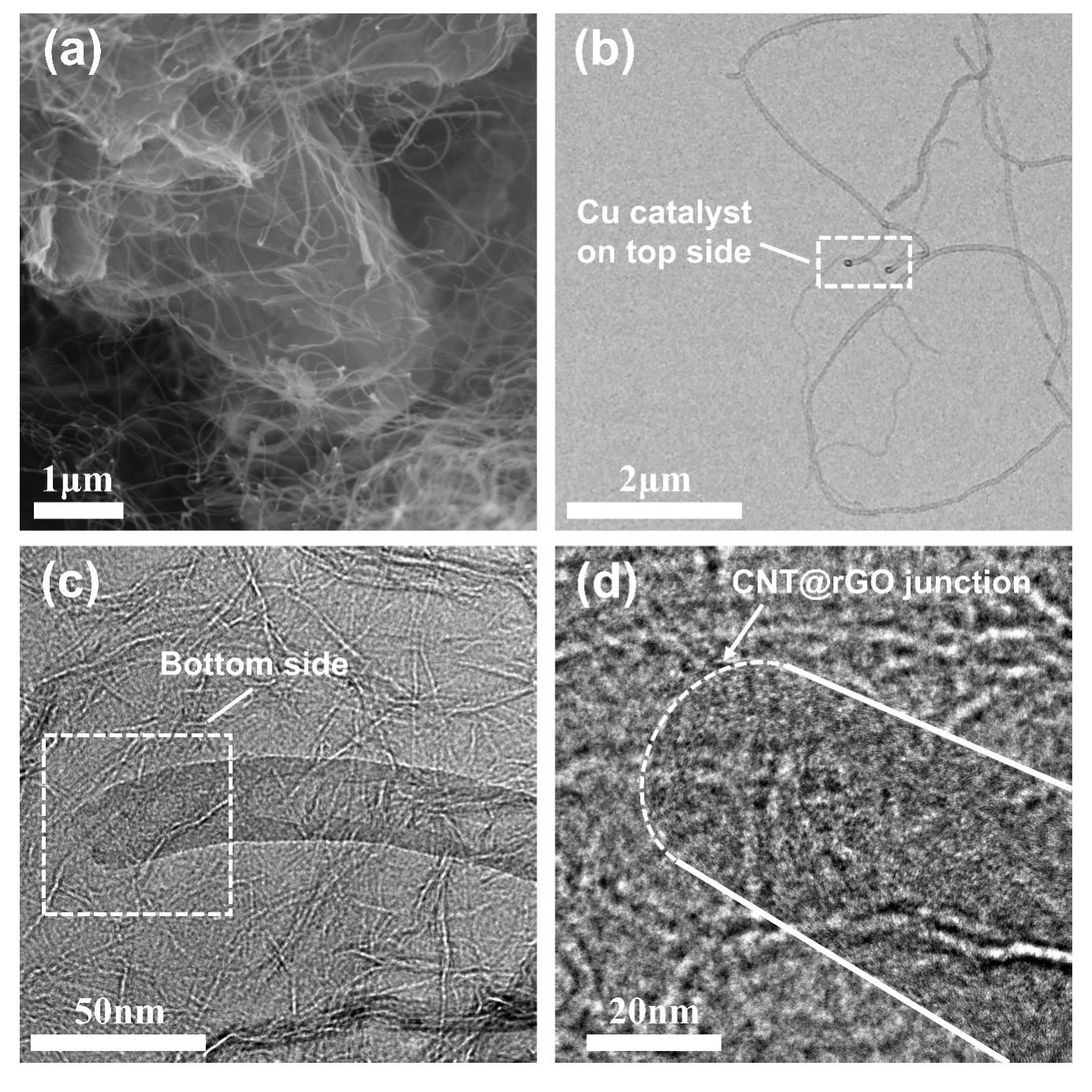


**Figure S1.** CNT@rGO images of each region at different magnifications (a) CNT@rGO three-dimensional structure; (b) HRTEM image of the tip of a carbon nanotube; (c) HRTEM image of the bottom of a carbon nanotube; (d) HRTEM image of CNT@rGO junction.


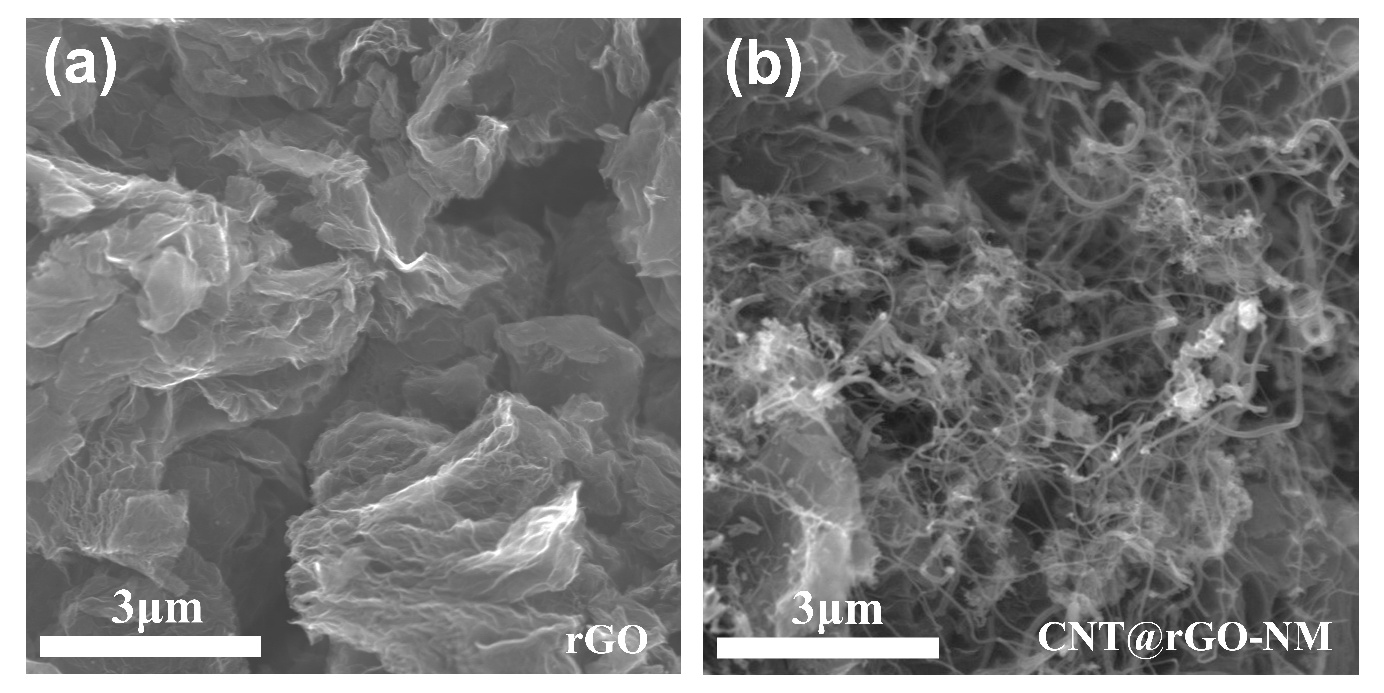


**Figure S2.** SEM images of (a) rGO; (b) CNT@rGO-NM.


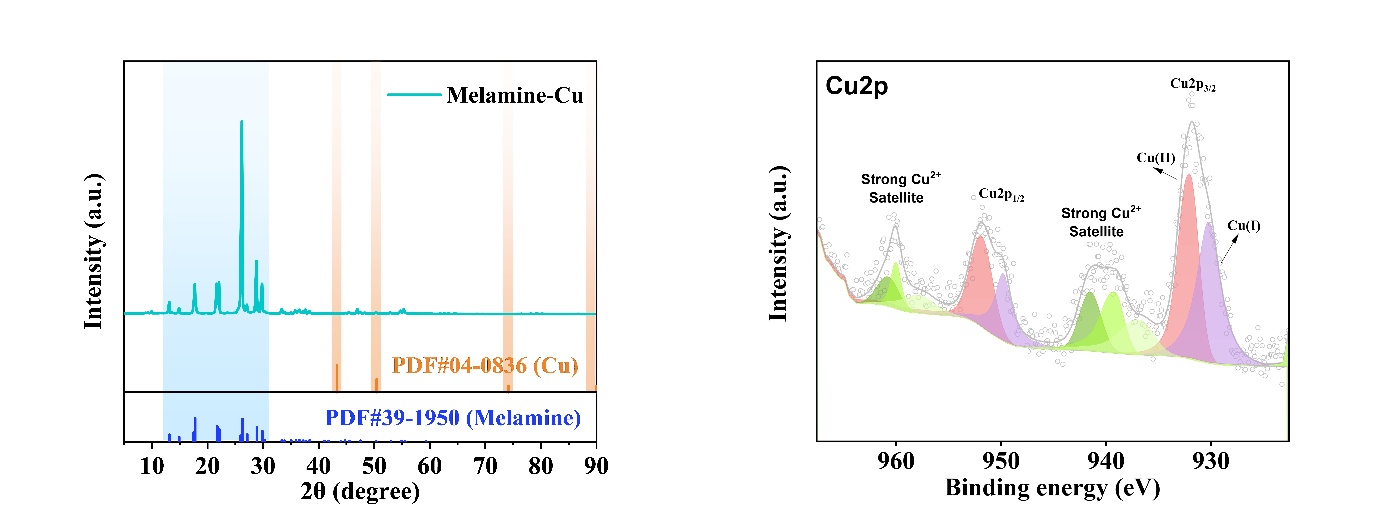
**Figure S3.** (a) XRD spectra of Melamine-Cu; (b) XPS spectrum of Cu2p for Melamine-Cu.

**
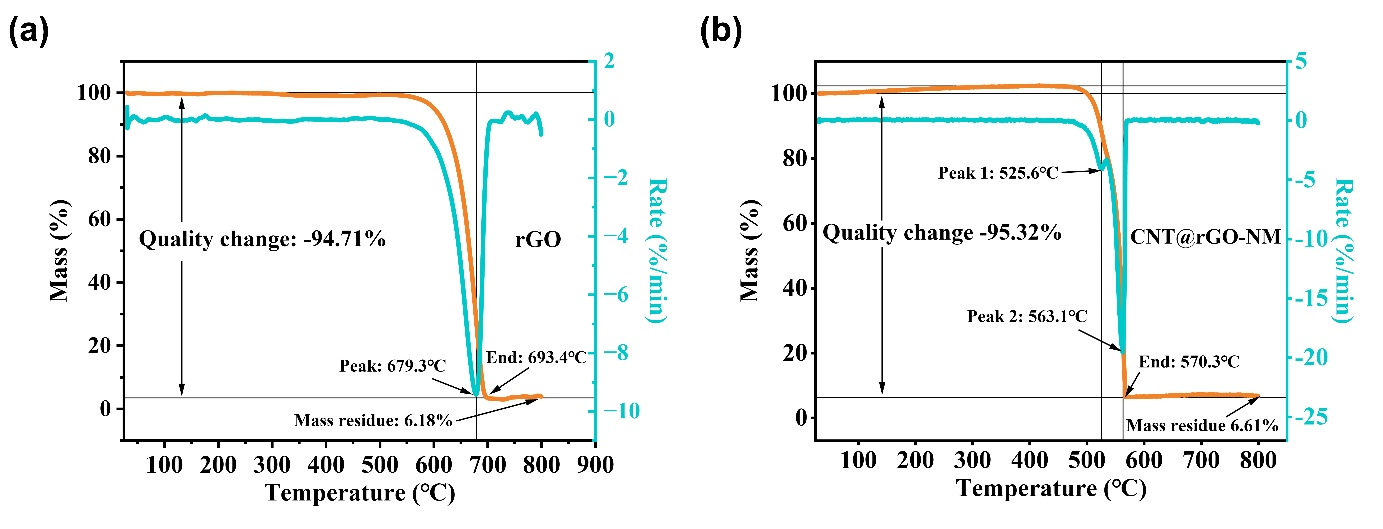
**

**Figure S4.** TG and DTG curves of (a) rGO; (b) CNT@rGO-NM.


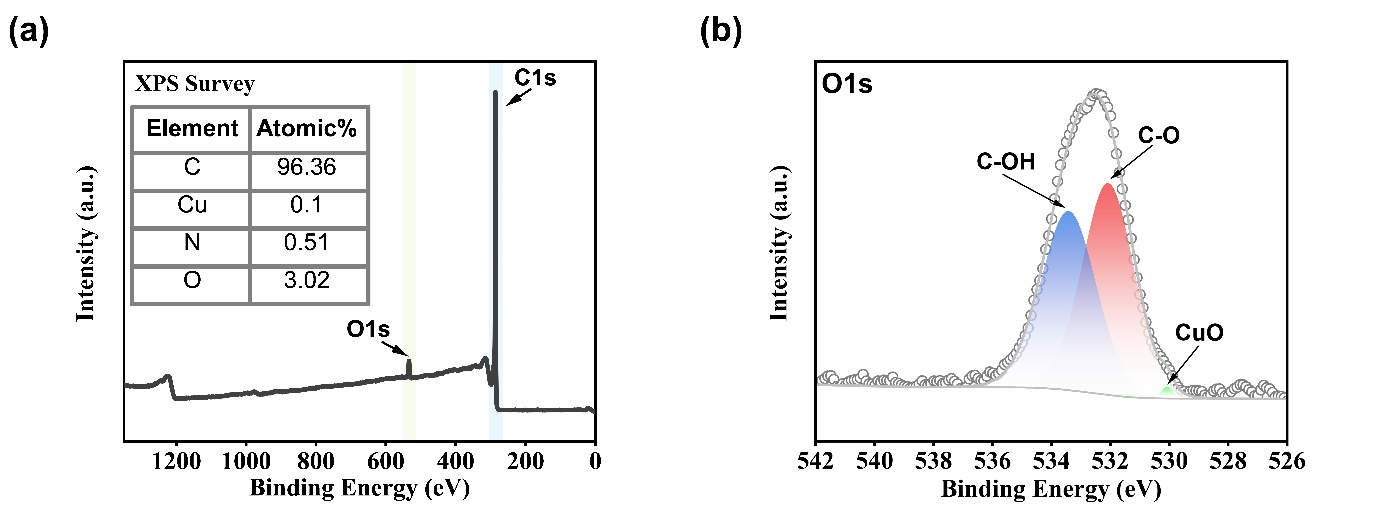


**Figure S5.** XPS characterization of CTN@rGO (a) total spectrum of CNT@rGO; (b) O1s spectrum of CNT@rGO.


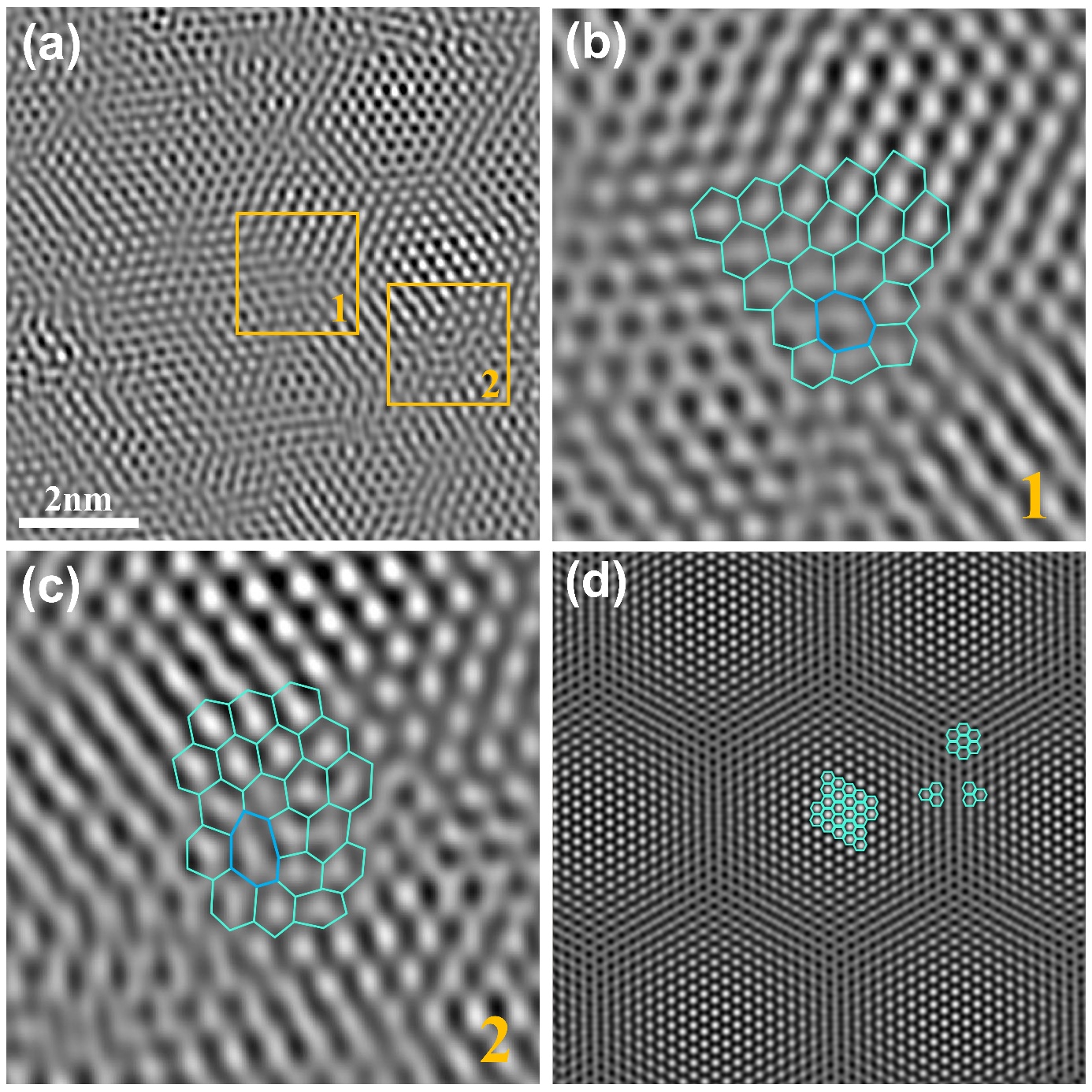


**Figure S6.** Defect analysis of the connection area (a) Noise-filtered BF-STEM image of the connection area; (b-c) magnified images of the 7-membered rings; (d) Simulated BF-STEM images of a twisted bilayer graphene with a twist angle of 3 degree. A defect-free moiré system doesn’t has 7-membered rings.


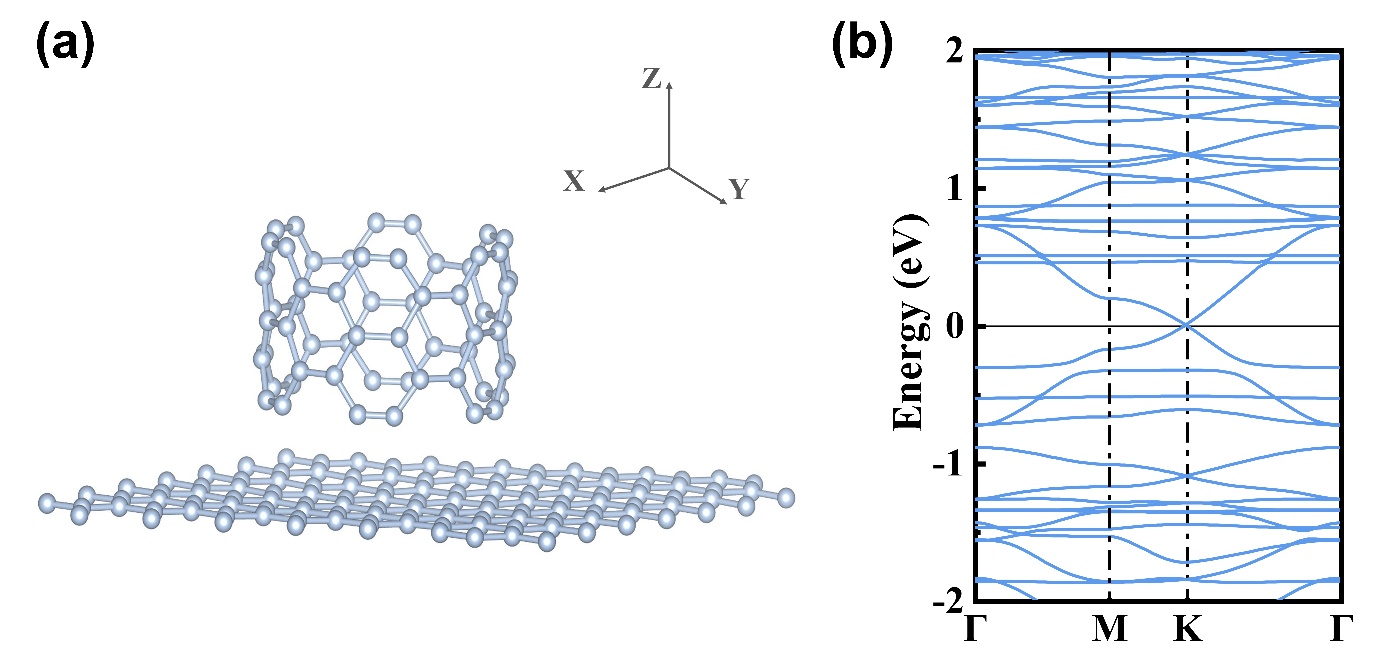


**Figure S7.** (a) Modeling of carbon nanotube–graphene van der Waals connection; (b) Band structure diagram of carbon nanotube–graphene van der Waals connection.


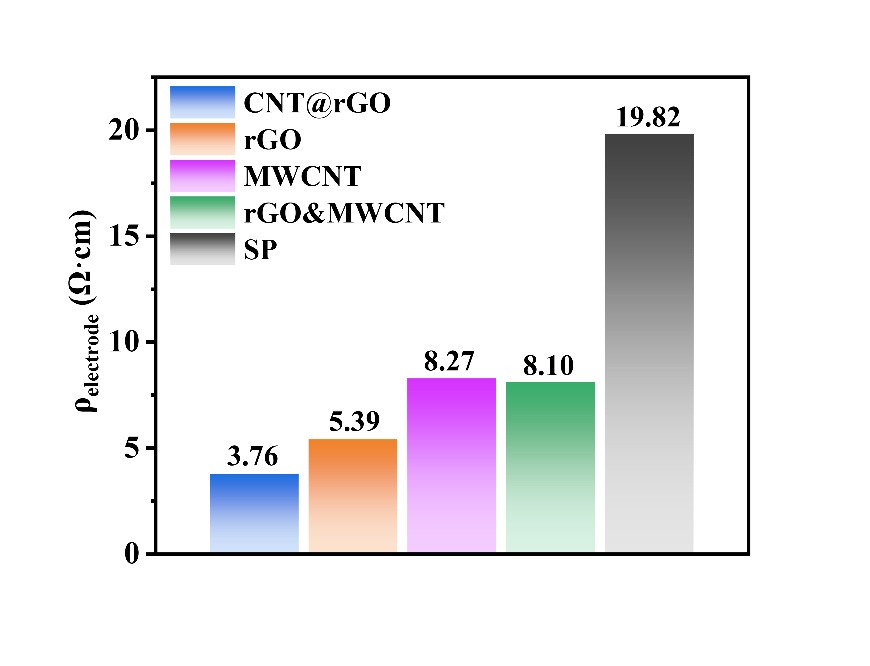


**Figure S8.** Pole piece resistivity for different electrodes.


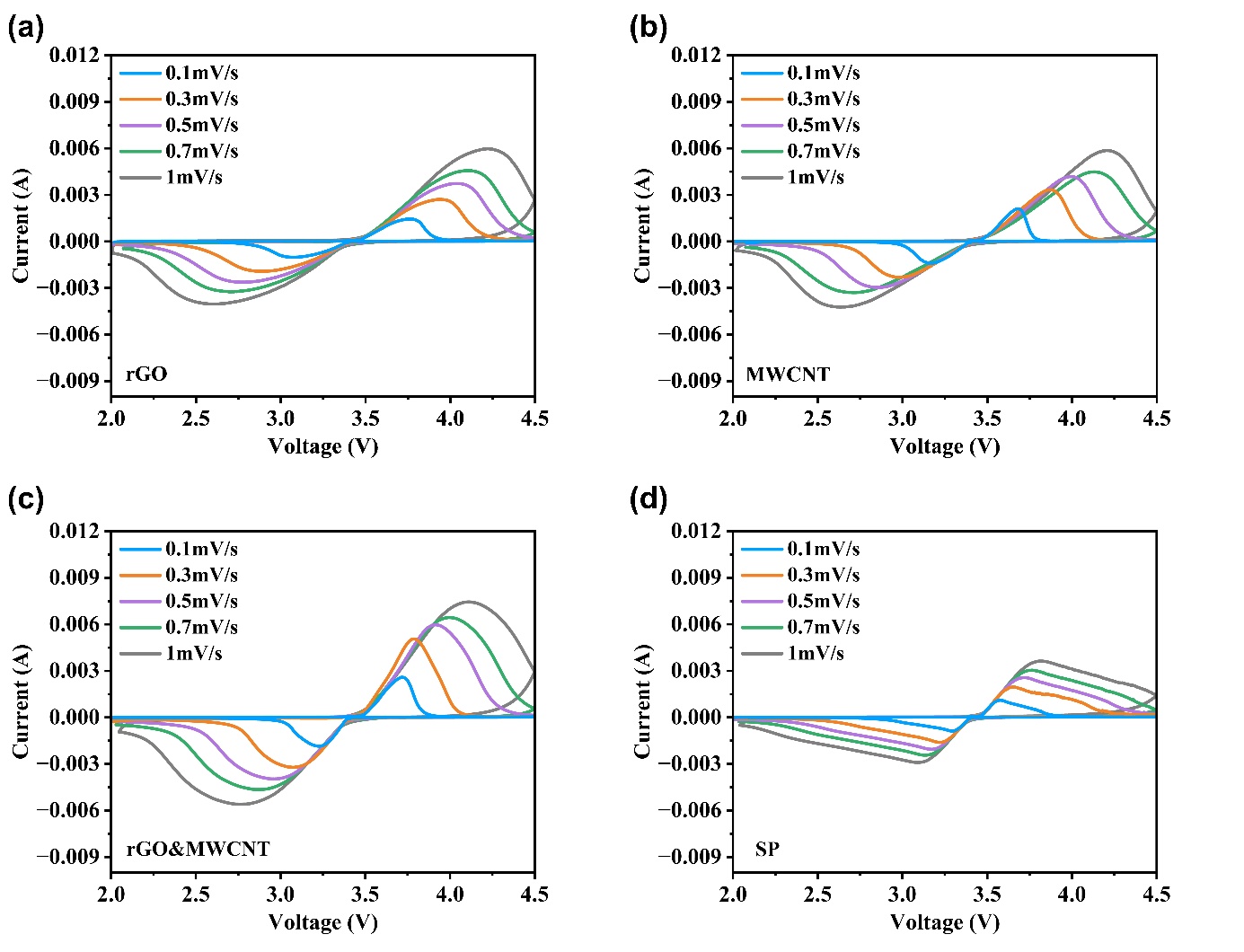


**Figure S9.** CV curves of varying sweep speeds for different electrodes (a) rGO; (b) MWCNT; (c) rGO&MWCNT; (d) SP.

**
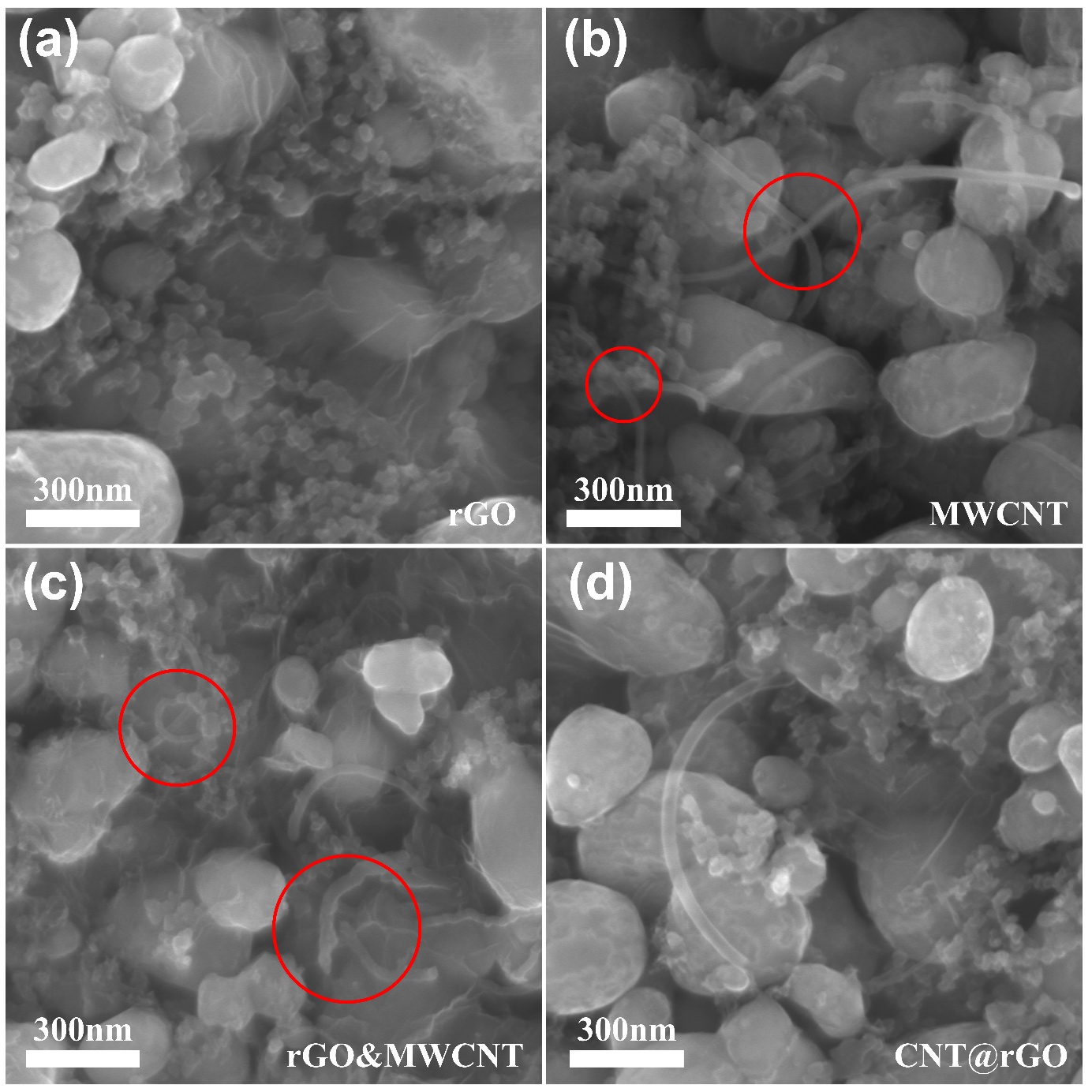
**

**Figure S10.** SEM images of the electrode cross section (a) rGO; (b) MWCNT; (c) rGO&MWCNT; (d) CNT@rGO.


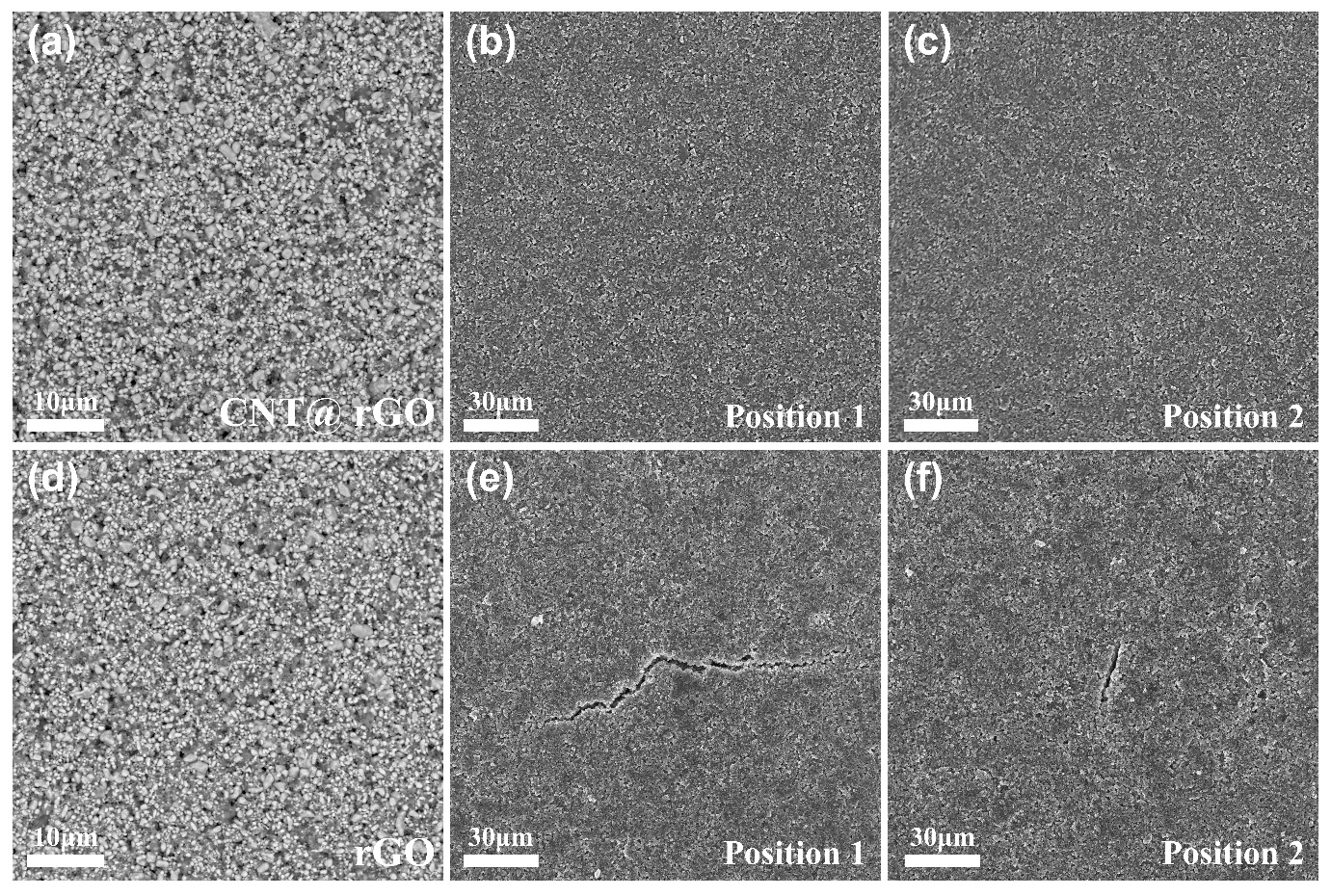


**Figure S11.** SEM images of (a) pristine CNT@rGO electrode; (b) after-cycling CNT@rGO electrode at Position 1; (c) after-cycling CNT@rGO electrode at Position 2; (d) pristine rGO electrode; (e) post-cycling rGO electrode at Position 1; (f) post-cycling rGO electrode at Position 2.


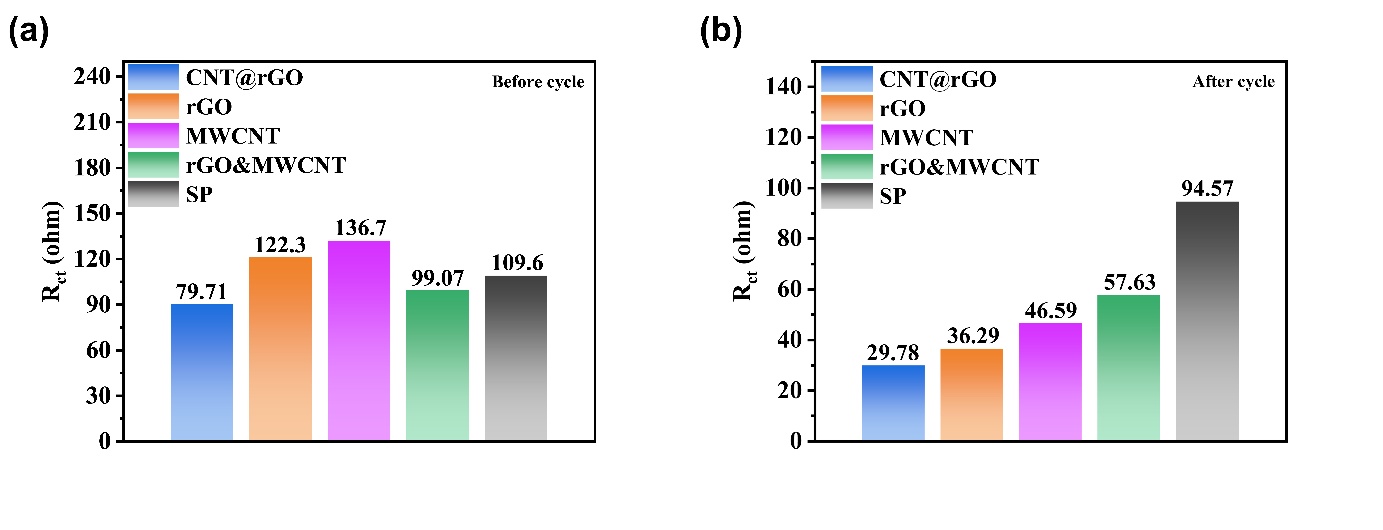


**Figure S12.** R_ct_ of electrode impedance for different electrodes (a) before cycle; (b) after cycle.


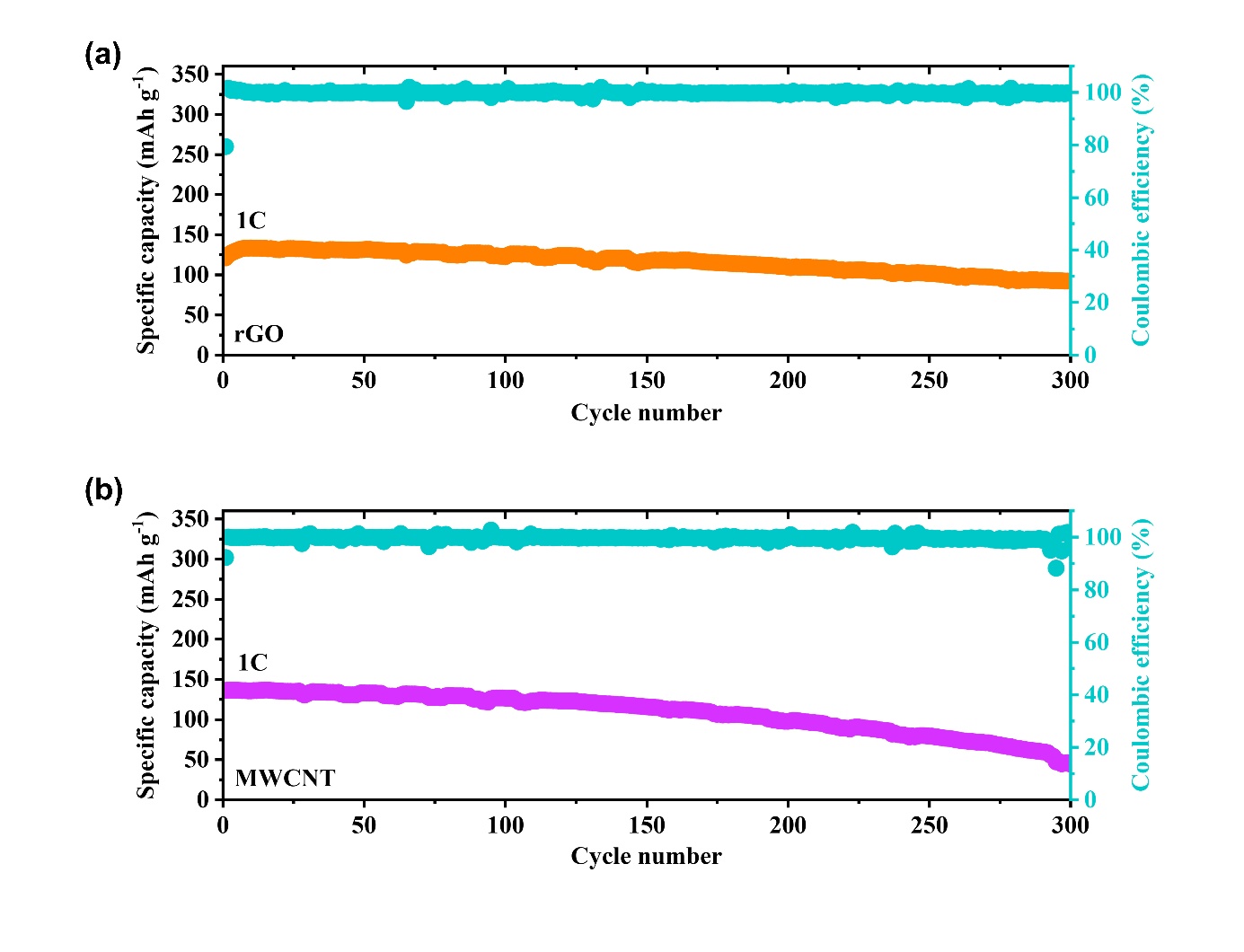


**Figure S13.** Long cycle test of electrodes with different conductive agents (a) rGO; (b) MWCNT.


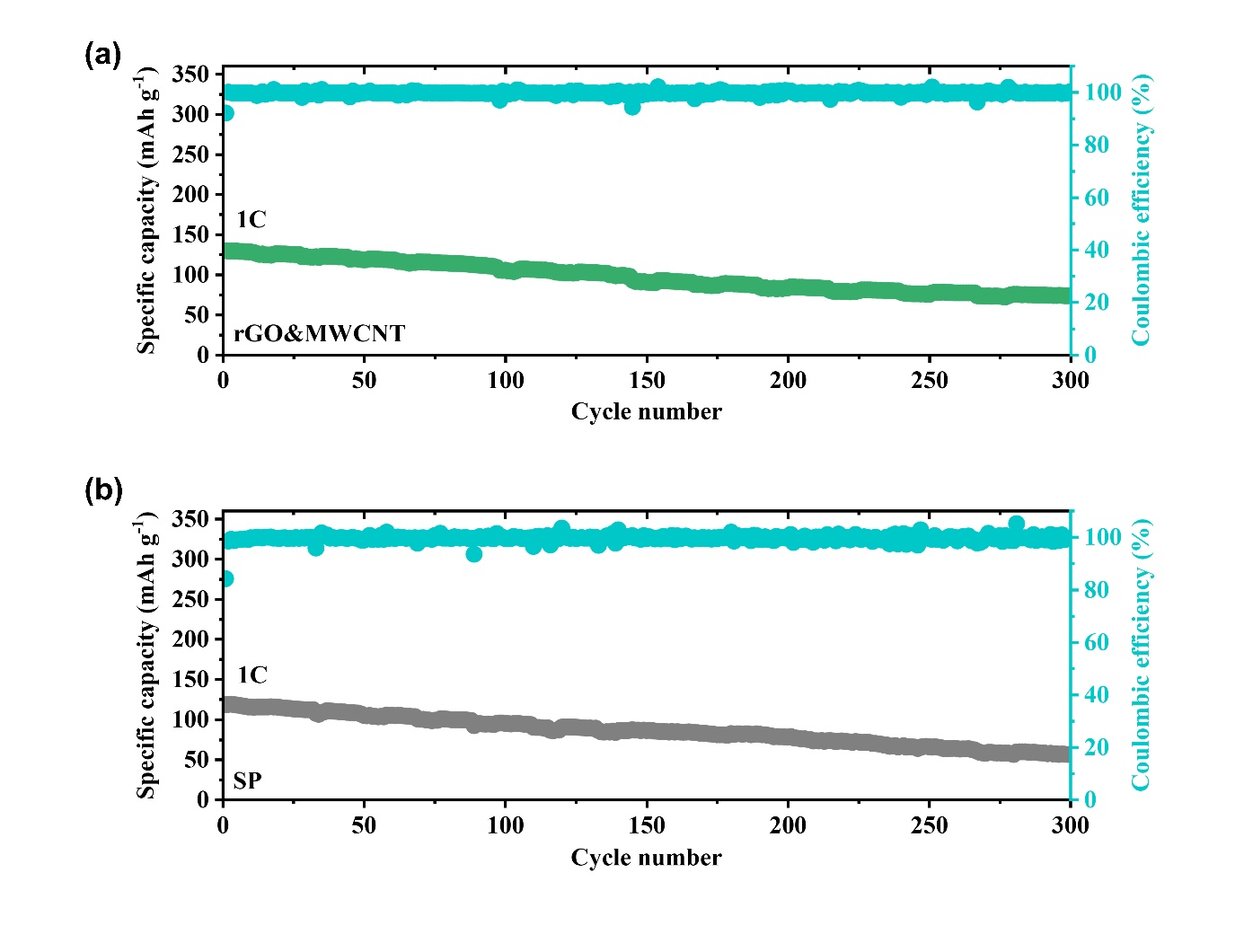


**Figure S14.** Long cycle test of electrodes with different conductive agents (a) rGO&MWCNT; (b) SP.


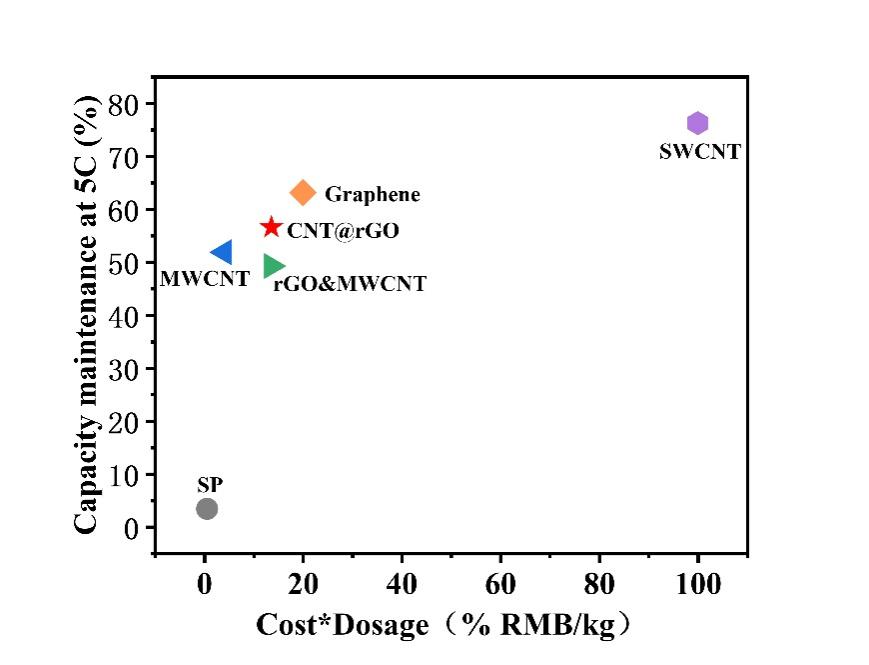


**Figure S15.** Compare the cost and performance of different conductive agents.
